# Meta Shack–Hartmann wavefront sensor with large sampling density and large angular field of view: phase imaging of complex objects

**DOI:** 10.1038/s41377-024-01528-9

**Published:** 2024-08-12

**Authors:** Gi-Hyun Go, Dong-gu Lee, Jaeyeon Oh, Gookho Song, Doeon Lee, Mooseok Jang

**Affiliations:** Department of Bio and Brain Engineering, Korea Advanced Institute of Science and Technology (KAIST), Daejeon, Republic of Korea

**Keywords:** Optical sensors, Metamaterials, Imaging and sensing

## Abstract

Shack–Hartmann wavefront sensors measure the local slopes of an incoming wavefront based on the displacement of focal spots created by a lenslet array, serving as key components for adaptive optics for astronomical and biomedical imaging. Traditionally, the challenges in increasing the density and the curvature of the lenslet have limited the use of such wavefront sensors in characterizing slowly varying wavefront structures. Here, we develop a metasurface-enhanced Shack–Hartmann wavefront sensor (meta SHWFS) to break this limit, considering the interplay between the lenslet parameters and the performance of SHWFS. We experimentally validate the meta SHWFS with a sampling density of 5963 per mm^2^ and a maximum acceptance angle of 8° which outperforms the traditional SFWFS by an order of magnitude. Furthermore, to the best of our knowledge, we demonstrate the first use of a wavefront sensing scheme in single-shot phase imaging of highly complex patterns, including biological tissue patterns. The proposed approach opens up new opportunities in incorporating exceptional light manipulation capabilities of the metasurface platform in complex wavefront characterization.

## Abstract (web summary)

We developed a metasurface-enhanced Shack-Hartmann wavefront sensor with an unprecedented sampling density, achieving single-shot phase imaging of highly complex patterns.

## Graphical abstract

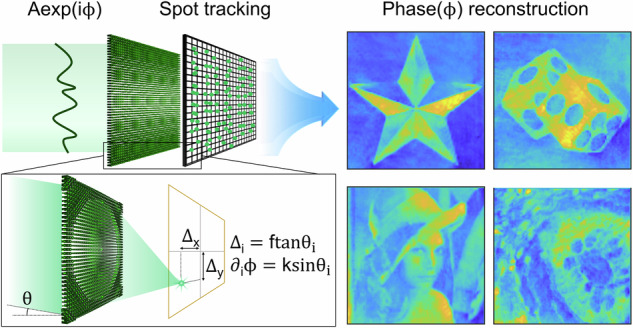

## Introduction

The importance of optical phase measurement has been increasingly recognized in a wide range of optical applications, including optical metrology, adaptive optics, biomedical imaging, and LiDAR technology^1–3^. A two-dimensional (2D) phase map is particularly useful in retrieving the surface topography and the morphology of microscopic objects. However, the direct measurement of the optical phase still remains a challenge because the oscillation frequencies of light waves are around 10^14^ Hz, which is much higher than the response speed of optoelectronic devices ranges up to 10^8^ Hz.

The current phase imaging techniques can be broadly divided into two categories: interferometry-based and computational phase retrieval methods. In the interferometry-based method, two beam paths-one for signal (object) and one for the reference- are commonly employed. The signal beam illuminates the object or sample, while the reference beam usually bypasses the sample entirely, resulting in a path difference to make interference patterns on a sensor plane. Consequently, the interferometry-based methods directly convert measured interference patterns into a phase map of an incoming beam via holographic reconstruction, providing superior phase measurement accuracy and large space-bandwidth product. However, they require bulky interferometry set-ups with a reference arm and are sensitive to even small fluctuations in wavelength scale^4–7^. To overcome those difficulties, many computational phase retrieval methods have been proposed, including transport-of-intensity equation^8^, ptychographic scanning methods^9,10^, and iterative algorithms^11–16^. However, the computational methods typically introduce various constraints such as the requirement of weak scattering samples and the need for multiple measurements, limiting their use in general high-speed and real-time phase measurement applications^17,18^.

Alternatively, wavefront sensing techniques can be considered as an indirect way to retrieve optical phase maps. A wavefront of light is a surface over which light waves have the same phase, and the light propagates perpendicular to the wavefront. Therefore, wavefront sensing techniques typically measure the propagation direction where an intensity pattern (e.g., focal spot) is captured at the Fourier plane. The displacement of the pattern can then be related to the incident wavefront angle and in turn to the optical phase gradient. Lastly, a 2D phase map can be retrieved by performing numerical integration of the optical phase gradient. In contrast to the conventional phase imaging techniques based on coherent light sources, wavefront sensing techniques are compatible with incoherent light sources as the amount of the intensity pattern displacement is irrelevant to an incoming wavelength. This feature enables its application in astronomical imaging, beam quality diagnosis, optical testing, fluorescence-based adaptive optical microscopy, and ophthalmology^19–24^. However, the spatial resolutions of typical wavefront sensing techniques, which are orders of magnitude lower than those of phase imaging techniques, have limited their use in characterizing slowly varying wavefront structures^25–30^.

Shack–Hartmann wavefront sensor (SHWFS) is the most widely used class of wavefront sensing methods. With its simple design composed of a lenslet array and an image sensor, it enables single-shot operation and exhibits excellent stability under various practical settings^31,32^. In principle, SHWFS can achieve 2D phase imaging for arbitrarily complex objects by integrating the local phase gradient values measured at each lenslet position. However, the lenslet array in traditional SHWFS is manufactured using MEMS fabrication technology with limited minimum feature size and maximum curvature of microstructures, resulting in the lenslets’ size in the range of ~100 μm with a low numerical aperture (NA)^27^. Therefore, the sampling density and the maximum measurable angle are respectively limited to around 100 per mm^2^ and 1°, which are not sufficiently high for quantitative phase imaging of complex phase objects. Because of those critical limits, conventional SHWFS has been only used to characterize slowly varying wavefront structures that can be expressed with low-order Zernike polynomials.

Here, we aimed to break this conventional limit to enable phase imaging with metasurface-enhanced SHWFS, so-called meta SHWFS. Based on inherent capabilities, compact design, low weight, and compatibility of metasurface platforms, several research efforts on metalens array have been reported, but their exploration has been confined to beam diagnosis, light field imaging, or multiphoton quantum source^33–38^. For the first use of meta SHWFS in single-shot phase imaging, we judiciously considered the interplay between the parameters of lenslets—focal length and size—and the performance of meta SHWFS—the maximum angular range, the number of resolvable angles, and the spatial resolution. Consequently, we implemented the meta SHWFS composed of 100 × 100 metalenses with a lenslet diameter of 12.95 μm and a focal length of 30 μm, corresponding to NA of 0.21. It facilitates the wavefront measurement with a high sampling density of 5963 per mm^2^ and a large acceptance angle of up to ±8°, representing 100× better spatial resolution and 10× larger phase gradient compared to traditional SHWFS systems. With these superior capabilities, we have demonstrated wide-angle position detection for an incoherent light source and phase imaging of complex objects synthesized with SLM, including confluent histopathologic tissue structure. We expect that meta SHWFS, with additional optical functionalities of the metasurface platform, would provide unprecedented opportunities in wavefront sensing and phase imaging.

## Results

### Design of meta SHWFS

**Fig. 1:**
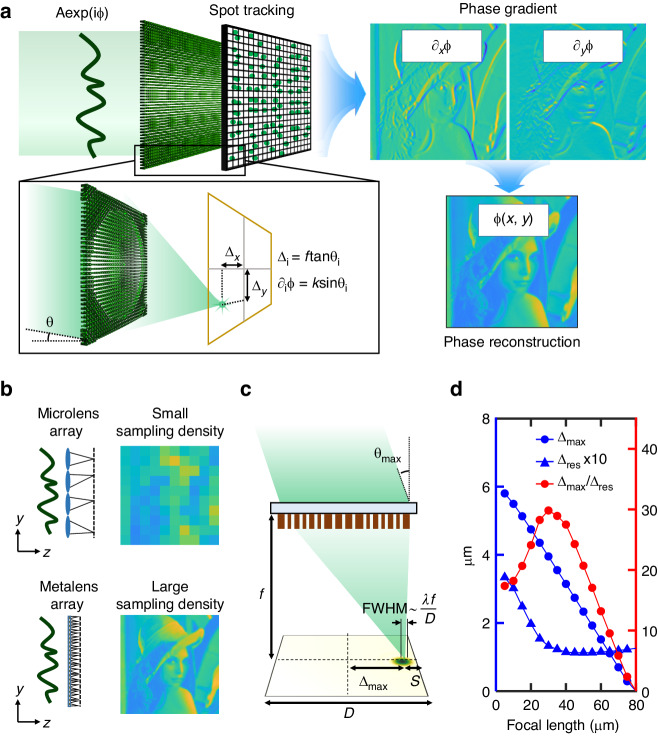
Design principle of meta Shack-Hartmann wavefront sensor. **a** Schematic of the meta SHWFS. **b** Effect of sampling density of SHWFS in phase reconstruction. **c** Illustration of a sampling unit of the meta SHWFS with related lenslet parameters. The maximum allowable displacement ∆_max_ is set with a minimum space S to take account of the finite width of the focal spot. **d** Maximum allowable displacement ∆_max_, localization accuracy ∆_res_, and their ratio ∆_max_/∆_res_ for different focal lengths. The ∆_max_/∆_res_ is maximized at the focal length of 30 μm

Figure 1a displays a schematic image of the meta SHWFS, comprised of an array of metalenses. Similar to conventional SHWFS, an image sensor is placed at the focal plane of metalenses in the meta SHWFS. In this configuration, the focal spot displacement *Δ* can be related to the incident angle *θ* of the local wavefront on each lens, with $\varDelta =f\times \tan \theta$ where *f* is the focal length of the metalenses. Subsequently, the wavefront angle *θ* is related to the local phase gradient ∂*ϕ* by ∂*ϕ* *=* *k sin θ* where *k* is the magnitude of wavevector (i.e., $k=2\pi /\lambda$, where *λ* is wavelength). Therefore, the local phase gradient can be derived from the focal spot displacement on each lens so that the 2D phase information of the incoming beam can be retrieved by numerically integrating the local gradient (details in Supplementary Note 1 in Supplementary Information).

In general, the performance of SHWFS is defined by the three key parameters—the maximum acceptance angle *θ*_max_, the number of resolvable angles (i.e., the number of resolvable levels of phase gradient) *N*_*θ*_, and the sampling density *N*_l_. To avoid the cross-talk between neighboring lenses, the incident angle *θ* should be set within the angular range where the corresponding displacement *Δ* does not exceed the boundary of each lens (i.e., [−*D*/2, *D*/2] where *D* is the size of each meta lens). Therefore, *θ*_max_ is roughly set as ${\tan }^{-1}\left(D/2f\right)$. On the other hand, *N*_*θ*_ is given as the number of resolvable spot positions within the region of each lens. Because the resolvable spot displacement is roughly given as the spot size, *N*_*θ*_ can be approximated to ${D}^{2}/{(\lambda /2{\rm{NA}})}^{2}$ where NA is expressed as $\sin \left[{\tan }^{-1}(D/2f)\right]$. Finally, *N*_1_ is simply proportional to 1/*D*^2^ as each lens locally samples the phase gradient information at a point.

The metalens diameter is the key parameter that determines the sampling density of meta SHWFS, as illustrated in Fig. 1b. For phase imaging applications, we aimed to fabricate the diameter of metalens, *D*, in the range of 10 µm, which is ten times smaller than that of conventional SHWFS. In this study, we determined the metalens diameter *D* as 12.95 μm to be the least common multiple of the metasurface lattice size *U* = 0.35 μm and the effective pixel size of *P* = 0.4625 μm, aiming for high sampling density with easy alignment. The effective pixel size of 0.4625 μm, which is comparable to the pixel size of recently developed CMOS sensors, was accomplished through a 4× imaging system with a physical pixel size of 1.85 μm (FLIR Blackfly S BFS-U3-120S4M-CS). It should be noted that *D* cannot be set to an arbitrarily small value because the undesired diffraction effect significantly affects the focusing quality, such as spot-to-background contrast, when the diffraction-limited spot size gets closer to the size of the lens itself.

Along with the lenslet diameter *D*, the focal length of the metalens *f* serves as a crucial parameter that determines *θ*_max_ and *N*_*θ*_. In our configuration with a small *D*, it is particularly challenging to achieve practically usable *N*_*θ*_ as *N*_*θ*_ has at least a power-of-two dependence on *D*. For phase imaging, *N*_*θ*_ needs to be sufficiently high to resolve various levels of phase slopes presented in complex structures like biological objects. Although, in the general relation, *θ*_max_ and *N*_*θ*_ monotonically increase with decreasing *f*, the ratio of the imaging pixel size *P* to the focal spot size should be taken into consideration in a practical scenario with a finite pixel size of an image sensor. If the spot size is smaller than a single pixel, it becomes impossible to accurately localize the spot for a subpixel displacement. Conversely, when the spot size is significantly larger than a single pixel, the presence of numerous noise sources, such as shot noise and dark noise, hinders the accurate tracking of the centroid position.

Therefore, we determined the optimum value of focal length considering the spot localization errors at various signal-to-noise levels. First, we defined the maximum allowable displacement of the focal spot *Δ*_max_ more accurately with a minimum space *S* to take account of the finite width of the focal spot (i.e., ${\varDelta }_{\max }=D/2-S$). As shown in Fig. 1c, we set *S* to be twice the full width at half maximum (FWHM), ~*λ*/(2NA), to ensure that more than 90% of the focal intensity is distributed within the corresponding region of a lenslet. Then, we assessed the root mean square of localization error, *Δ*_res_, for various focal lengths and signal-to-noise ratios (SNR) (details in Supplementary Note 2 in the Supplementary Information). For this purpose, we determined the position of the simulated focal spot through the calculation of radial symmetry with subpixel accuracy, closely approaching the theoretical lower limit of the error as defined by the Cramér-Rao bound (details in Supplementary Note 3 in the Supplementary Information)^39^. With *Δ*_max_ and *Δ*_res_, the maximum acceptance angle and the resolvable angle can be determined through the relation $\varDelta =f\times \tan \theta$ so that *N*_*θ*_ is given as ${\left(2{\varDelta }_{\max }/{\varDelta }_{{\rm{res}}}\right)}^{2}$ with the small-angle approximation (i.e., $\theta\,\lesssim\,{20}^{\circ}$). Figure 1d shows *Δ*_max_, *Δ*_res_, and ${\varDelta }_{\max }/{\varDelta }_{{\rm{res}}}$ with SNR = 10 dB. We determined the focal length to be 30 μm to maximize the value of ${\varDelta }_{\max }/{\varDelta }_{{\rm{res}}}$ (details in Supplementary Note 2 in the Supplementary Information). We note that the focal length can be set shorter to improve *Δ*_max_ at the price of *N*_*θ*_ for certain applications where *Δ*_max_ serves as a critical parameter. Consequently, each metalens can accommodate *θ*_max_ of 8°, which is 10 times larger than that of the conventional SHWFS, with the large degrees of freedom in measuring wavefront angle, *N*_*θ*_ ~ 3600. From the principle of meta SHWFS, we can derive the equation $\varDelta {\phi }_{{\rm{res}}}/D=2{\rm{\pi }}/{\rm{\lambda }}\times \sin ({\tan }^{-1}{\varDelta }_{{\rm{res}}}/f)$, where *D* is the metalens diameter ($D\,=\,12.95\,{\rm{\mu }}{\rm{m}}$), *f* is the focal length ($f=30\;{\rm{\mu }}{\rm{m}}$), and *Δ*_res_ is the localization accuracy (${\varDelta }_{{\rm{res}}}\,=\,0.13\,{\rm{\mu }}{\rm{m}}$ for SNR = 10 dB, details in Supplementary Note 2). Therefore, we can determine the accuracy of the wavefront measurement as $0.1\lambda$. It is noteworthy that, leveraging the flexibility in setting lens parameters using a metasurface platform, the meta SHWFS overcomes the longstanding limit in spatial resolution and acceptance angle in conventional SHWFS while achieving a sufficiently large value of *N*_*θ*_ for phase imaging.

### Large angle calibration of meta SHWFS

**Fig. 2:**
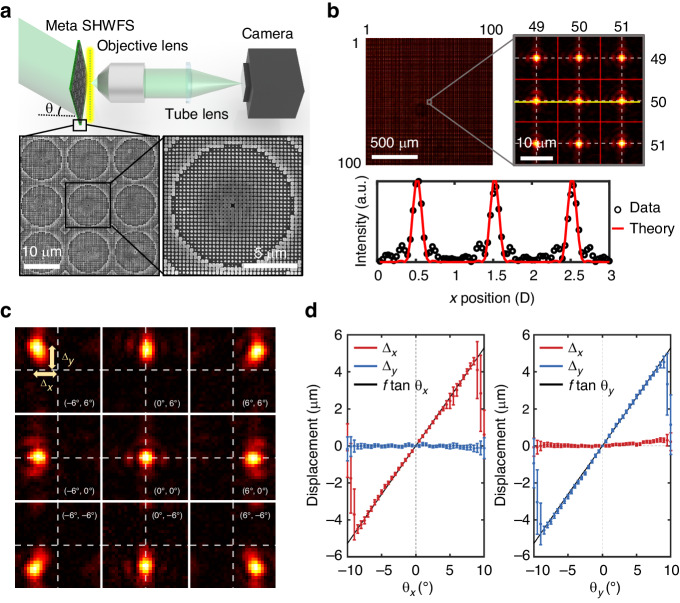
Large angle calibration of the meta SHWFS. **a** Experimental setup for large angle calibration and the SEM image of the fabricated metalenses. **b** Focal intensity distribution created by the metalens array. The numbers outside the image denote the order of the metalenses. White dotted lines indicate the central lines of each metalens. The lower plot depicts the intensity profile along the yellow line. **c** Focal spot behaviors of a central metalens for different angles of incidence, following the equation ∆_*i*_ = *f* tanθ_*i*_ (*i* = *x*, *y*). **d** Focal spot displacements for various incident angles. The 100 × 100 focal spots uniformly move together as the incident angle changes. The large fluctuations in spot localization for the large angles beyond 8° is attributed to cross-talk between the focal spots of adjacent metalenses

The lenslet array in our meta SHWFS, composed of 100 × 100 metalenses, shares the same working principle with dielectric metasurfaces reported in previous studies^29,40–46^. Metalenses consisted of silicon nitride (SiN_*x*_) rectangular cuboids arranged on a subwavelength square lattice with a periodicity of *U* = 350 nm. The width of each meta-atom was precisely controlled within a range from 60 nm to 275 nm to achieve 2π phase coverage at a wavelength of 532 nm with a height of 630 nm (details in Supplementary Note 4 in the Supplementary Information). The phase values at the position of each meta-atom were sampled from the hyperbolic phase profile for a converging spherical wave: ${\psi }_{{\rm{lens}}}\left(x,y\right)=-\frac{2\pi }{\lambda }\left(\sqrt{{f}^{2}+{x}^{2}+{y}^{2}}-f\right)$ where *x* and *y* are the coordinates of the meta-atoms. Each metalens, with the size of *D* = 12.95 μm, was comprised of 37 × 37 meta-atoms. The scanning electron microscopy (SEM) image of the fabricated metalenses is shown in Fig. 2a. The detailed fabrication procedures are provided in Supplementary Note 5 of the Supplementary Information.

To characterize the meta SHWFS, we measured the behaviors of focal spots for varying incidence angles in the configuration where each metalens corresponds to the sensor area of 28 × 28 pixels, as shown in Fig. 2a. The meta SHWFS can be accurately aligned by observing the focal spots of the metalenses at four corners (details in the Supplementary Note 6 of the Supplementary Information). The incidence angle of a plane wave was controlled using two galvanometer mirrors (omitted in Fig. 2a). Figure 2b presents the intensity distribution of the focal spots at normal incidence. The FWHM of the focal spot profile is measured to be 1.80 µm, which is consistent with the theoretical expectation based on the NA of the metalenses and the imaging system, 0.21 and 0.16, respectively. Also, the diffraction pattern of the rectangular aperture appeared along horizontal and vertical directions. This diffraction effect becomes more significant for a smaller diameter, implying that there is a fundamental limit in reducing *D*. The focusing efficiency of our metalens was experimentally measured to be 47.7%. Here, the focusing efficiency is defined as the fraction of the light in the focal plane with a radius equal to three times the FWHM of the focal spot^42^. Figure 2c shows the focal intensity distribution at the central metalens for various incident angles. Although the focal spots present slight deformation due to off-axis aberration for an incidence angle larger than 5°, the symmetricity of the peaks and the peak-to-background ratio were sufficiently high to track their position with subpixel accuracy through the calculation of radial symmetry center as shown in Fig. 2d (details in Supplementary Note 3 in the Supplementary Information)^39^. Within the angular range of −8° < *θ* < 8°, the focal spots were shown to be uniformly translated following the relation $\varDelta\,=\,f\,\times\,\tan \theta$ with small standard deviations, breaking the limit of conventional SHWFS in the maximum acceptance angle *θ*_max_. The large fluctuations in spot localization for the large angles beyond 8° are attributed to cross-talk between the focal spots of adjacent metalenses.

### Three-dimensional (3D) position tracking of incoherent light source

To verify the capability of the proposed meta SHWFS for an incoherent light source, we demonstrated 3D position tracking of a light-emitting diode (LED) with a size of 0.3 mm and an emission spectrum of 520–535 nm. Since our metalens is designed for 532 nm, chromatic aberration can occur. This aberration arises from dispersion within the periodic lattice, akin to Fresnel lenses, resulting in different focal lengths given by $f\,=\,{f}_{{\rm{c}}}{\lambda }_{c}/\lambda$, where *f*_c_ is the focal length of 30 μm and *λ*_c_ is the center wavelength of 532 nm, respectively. Due to the extremely short focal length of our metalens, the shift in focal length is limited to a few micrometers, even across the wide wavelength range from 432 nm to 632 nm. Because this change in focal distance is smaller than the depth of field, ${\rm{DOF}}\,=\,\lambda /\left[2\left(1\,-\,\cos \theta \right)\right]\,=\,11.8\,{\rm{\mu }}{\rm{m}}$, the impact of achromatic aberration remains marginal, enabling the use of the proposed meta SHWFS in the entire visible spectrum of 432–632 nm (details in Supplementary Note 7 in the Supplementary Information). To achieve better accuracy in a wider wavelength range, one can employ achromatic metalens^33,34,47^. The spatial coherence of the LED should be considered for the measurement situation of meta SHWFS. Without spatial coherence, the wavefront cannot be determined because the phase relationship between different parts of the wavefront varies randomly. The Van Cittert–Zernike theorem implies that the wavefront from an incoherent source appears coherent at a large distance. This theorem provides a condition for wavefront measurement: ${D}_{{\rm{coh}}}=4\lambda L/{\rm{\pi }}{D}_{{\rm{LED}}}$, where *D*_coh_ is the size of the coherence area over which the wavefront can be considered spatially coherent, *L* is the distance from the LED to the metalens array, and *D*_LED_ is the size of the LED. In our experiments, the LED size was 0.3 mm, and the meta SHWFS size was 1.295 mm. Therefore, for a distance larger than the distance of 2.3 mm where the coherence area on the metalens surface matches the size of meta SHWFS, the proposed sensing scheme can be applied.

**Fig. 3:**
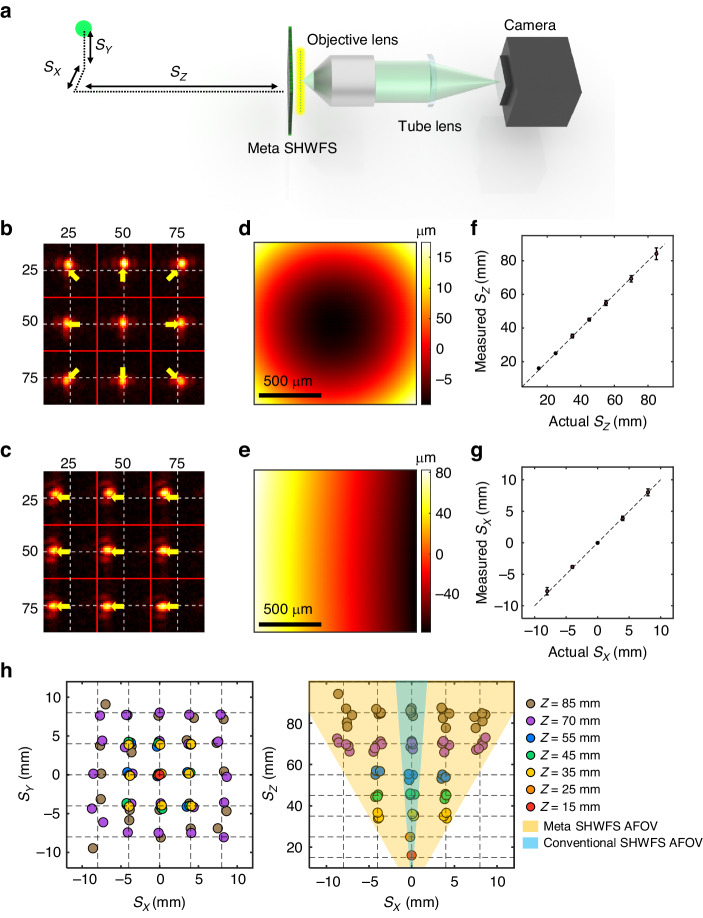
3D position tracking of incoherent light source. **a** Experiment setup to demonstrate position tracking of a LED. **b**, **c** The behaviors of focal spots at (*S*_*X*_*, S*_*Y*_*, S*_*Z*_) = (0, 0, 15) mm and (*S*_*X*_*, S*_*Y*_*, S*_*Z*_) = (8, 0, 70) mm, respectively. **d**, **e** Wavefronts reconstructed by the focal spot displacements, presented in (**b**, **c**), respectively. **f**, **g** Measured LED positions versus actual positions. The LED positions were determined by quadratic fitting of the reconstructed wavefronts. **h** Distribution of measured LED positions in the *x*–*y* plane (left) and *x*–*z* plane (right), respectively. The dotted grid represents the actual position of the LED. Note that the sensing area of meta SHWFS is much larger than that of conventional SHWFS

Figure 3a illustrates the schematic of the experimental setup. We note that similar configurations based on SHWFS have been preferred over other interferometric methods in the position tracking and alignment of optical components and beam quality assessment, especially when dealing with LED-based optical systems. Figure 3b, c shows the focal intensity distribution for the LED located at (*S*_*X*_*, S*_*Y*_*, S*_*Z*_) = (0, 0, 15) mm and (8, 0, 70) mm, respectively. The radial shifts of the focal spots were more prominent when the LED got closer to the meta SHWFS, while the transverse shifts became more pronounced when the LED was translated in *x*- or *y*-directions. From the displacements of the 100 × 100 focal spots, we reconstructed the wavefront *w* (*x*, *y*) by integrating the gradient of wavefront, $\sin ({\tan }^{-1}\frac{\varDelta }{f})$, as shown in Fig. 3d, e. As shown in Fig. 3e, the measurable total wavefront change over the aperture was larger than 150 µm which corresponds to ~300λ. The incoming wavefront from the LED located at (*S*_*X*_*, S*_*Y*_*, S*_*Z*_) is expected to have a quadratic form as:

1 $$w\left(x,y\right)\,=\,\sqrt{{\left(x-{S}_{X}\right)}^{2}+{\left(y-{S}_{Y}\right)}^{2}+{{S}_{Z}}^{2}}\,\approx\,\frac{1}{2{S}_{Z}}\left[{\left(x-{S}_{X}\right)}^{2}+{\left(y-{S}_{Y}\right)}^{2}\right]\,+\,{const}$$

Therefore, the LED position (*S*_*X*_*, S*_*Y*_*, S*_*Z*_) was determined through quadratic fitting of the measured wavefront. Figure 3f, g illustrate that the meta SHWFS accurately detects the LED position for various positions. Figure 3h shows the measured LED positions in the *x*–*y* plane (left) and *x*–*z* plane (right). The dotted grid lines represent the real position of the LED light source. Exploiting the large acceptance angle, the angular field of view of meta SHWFS is extended to the range that is 10× larger than that of conventional SHWFS. The observed lower accuracy for *S*_*Z*_ value above 85 mm is attributed to the small aperture size, 1.295 mm, of the entire meta SHWFS. As expected, at *S*_*Z*_ = 85 mm, the maximum incidence angle near the edge of the aperture, ~0.44° $(={\tan }^{-1}(\frac{1.295}{2\times 85}))$, is in the comparable range with the resolvable angle of our meta SHWFS, ~0.27° (= 8°/30). This axial range can be extended up to ~10 cm with large-area metasurfaces that can be fabricated by novel lithography techniques in cost-effective manner^48–52^.

### Implementation of phase imaging using meta SHWFS

**Fig. 4:**
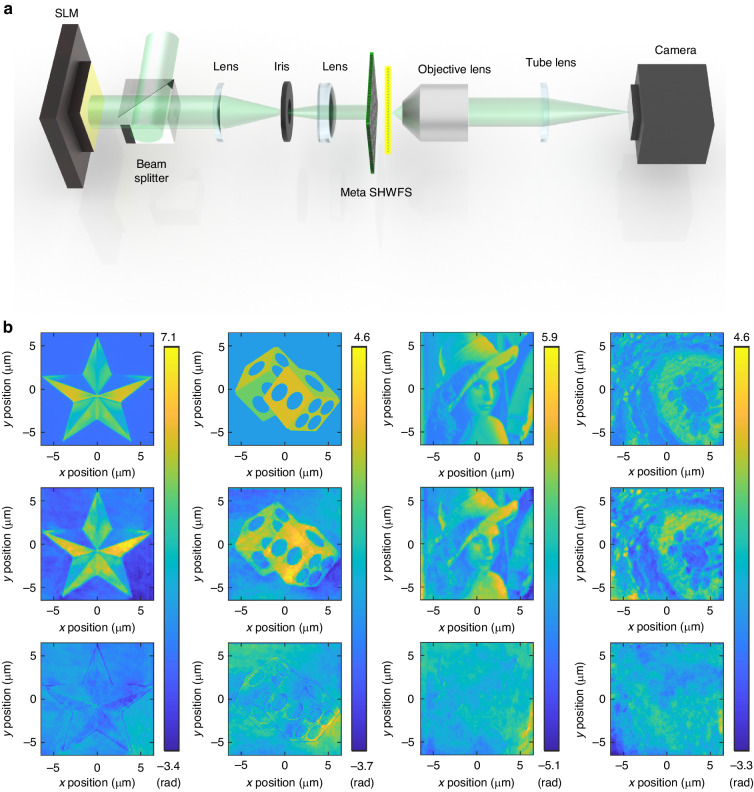
Demonstration of phase imaging using meta SHWFS. **a** Experiment setup for phase imaging via meta SHWFS. **b** Result of phase imaging. The first, second, and third rows represent the ground truth image (input image of SLM), the reconstructed phase, and the difference between them, respectively

Finally, we validated the phase imaging capability of the meta SHWFS. The phase imaging targets were synthesized using a spatial light modulator (SLM), as shown in Fig. 4a. The modulated phase pattern was directly projected on the input surface of the meta SHWFS through a 4*f* relay system with a spatial filter to get rid of undesired diffraction patterns from the SLM. The phase images were obtained from the measured phase gradient via a global least squares minimizer developed for reconstructing surface from gradients^53,54^. We tested phase imaging capability with the four targets with varying degrees of complexity and appearance, shown in the first row of Fig. 4b. More specifically, the star and the dice images have sharp edges with constant backgrounds while the standard Lena and the biological tissue images have confluent structures. Such patterns share structural features found in diverse phase imaging applications for man-made and natural targets. The second and third rows represent the reconstructed images and the differences between the reconstructed images and the ground truth. Figure 4b shows that the proposed scheme successfully reconstructs the phase images for all cases of targets. It is remarkable that the reconstructed phase image retains the fine details of biological tissue patterns including cell bodies and connective components. The differences are derived by subtracting the reconstructed values from the ground truth after correcting for the effects of magnification, rotation, and translation using the correlation method to compensate for misalignment in the experiments. We observed that the difference is relatively large at the edges of the image, where the phase abruptly changes. The standard deviation of the differences for the entire image is 0.12*λ*, which is consistent with the theoretical prediction. Lastly, we confirmed that the large value of *N*_*θ*_ = 3600 played a critical role in resolving such details with the further analysis of the wavefront slopes presented in the biological tissue pattern and the resolvable angles of the meta SHWFS (see Supplementary Note 8 in the Supplementary Information).

## Discussion

In this study, we have presented a meta SHWFS composed of 100×100 metalenses with a high sampling density of 5963 per mm^2^ and a large acceptance angle of 8°, representing a 100× better spatial resolution and 10× larger angular range compared to traditional SHWFS systems. We confirmed that each metalens provides a large number of degrees of freedom (i.e., *N*_*θ*_ ~ 3600) for measuring wavefront slopes even with the extremely small lens size of *D* = 12.95 µm. With this superior performance, we have demonstrated the 3D position tracking of an LED source with a large angular field of view and the phase imaging of complex objects. The experimental error for phase imaging of complex objects is 0.12*λ*, which is consistent with the theoretical prediction. The calibration method of SHWFS would further help in correcting marginal errors that may exist in our alignment and fabrication procedures^55^. We expect that the meta SHWFS can be directly used for numerous applications where a traditional SHWFS plays a crucial role such as in the characterization and alignment of optical components and adaptive optics for astronomical and biomedical imaging.

Sampling density *N*_l_ of the SHWFS is mainly determined by the lens size as following the relation: ${N}_{{\rm{l}}} \sim 1/{D}^{2}$. Therefore, one can use the smaller lenses to increase the sampling density. However, it should be noted that D cannot be set to an arbitrarily small value because the undesired diffraction effect significantly affects the focusing quality, such as spot-to-background contrast, when the diffraction-limited spot size gets closer to the size of the lens itself. We examined the focal spot behaviors of metalens arrays for different lens sizes (details in Supplementary Note 9 in the Supplementary Information). For a given NA of 0.21, due to the low peak-to-mean ratio and large cross-talk effects, we identified the lower bound for the size of metalens, which is about 6 μm (Sampling density ~ 28,000 mm^−2^). Meanwhile, the acceptance angle *θ*_max_ is primarily determined by the NA of the lenslet because the focal spot displacement is confined within the range of a single lenslet given by $\tan {\theta }_{\max }\,=\,{\varDelta }_{\max }/f\,<\,D/2f\,=\,\tan\;({\rm{asin}}\left({\rm{NA}}\right))$. To identify the practical upper bound of NA (i.e., upper bound of acceptance angle), we should consider the camera pixel size. To ensure accurate tracking of the focal spot, the focal spot size should be comparable to the camera pixel size, expressed as: *λ*/2NA ~ *P*, where *P* is the camera pixel size. Therefore, the maximum acceptance angle is limited up to ${\theta }_{\max } \sim {\rm{asin}}(\lambda /P)$. Computational methods such as the advanced phase unwrapping algorithm and the global matching approach with adaptive spot would help further increase the acceptance angle^56,57^. It is noteworthy that the performance can improve even further by employing metalenses for a wide field of view^58,59^ or leveraging the multifunctionality of metasurface technology. Supplementary Note 10 presents the potential strategies to address the trade-off between lens size and dynamic range. Furthermore, polarization-sensitive metalenses can be used to yield anisotropic phase information of biological targets such as monosodium urate crystals from a gout patient^60,61^.

In general, the number of pixels on an image sensor roughly sets the upper bound for the degrees of freedom of SHWFS, which can be quantified as the product of the number of wavefront sampling positions (i.e., the number of lenslets) and the number of resolvable angles in each lens *N*_*θ*_. Since the first development of SHWFS, CMOS sensor technology has evolved towards larger pixel numbers with smaller pixel sizes, achieving pixel numbers larger than 10^8^ and a pixel size of less than 1 µm. However, due to the limitations in conventional microlens arrays, the development of Shack–Hartmann-type wavefront sensors has primarily focused on the direction of increasing measurement accuracy rather than increasing spatial resolution, thereby not fully exploring the possible design space supported by the recent developments in CMOS sensor technology. Here, we have implemented the SHWFS with 100 × 100 sampling positions and 3600 resolvable angles with a sampling period in the range of 10 µm, exploring the new design space supported by the metasurface platform and the advanced sensor technology. Within this new design space, we could achieve the first successful demonstration of microscopic phase imaging. In this study, we used the CMOS censor (BFS-U3-120S4M), which has 4000 × 3000 pixels with a size of 1.85 µm. Because 28 × 28 camera pixels were allocated per metalens, the maximum number of metalenses was limited to 142 × 107. This number can be significantly increased based on the development in sensor technology, potentially along with on-chip integration of meta SHWFS. For instance, with the adoption of superior camera sensors (e.g., Samsung’s ISOCELL HP3, 200 MP with a pixel size of 0.56 µm), it is indeed possible to implement a small form-factor quantitative phase imaging unit, which achieves the sampling points of 500 × 500 without additional imaging system.

Due to the increased interest for biomedical imaging, optical system characterization, and industrial inspections, many interesting approaches have been proposed for single-shot complex field measurements. For instance, the input field can be obtained from the output intensity, exploiting light mixing within turbid media described based on transmission matrix formalism or utilizing a thin diffuser to generate a speckle pattern with shift-invariant property based on memory effect^62,63^. Based on advanced fabrication techniques, it is also possible to determine the incident angle from the energy distribution on four camera pixels combined with an aperture placed at the intersection^26^. Notably, this work achieved a high sampling density of 9246 mm^−2^ and a large acceptance angle of 30° by leveraging the monotonic relationship between the incident angle and the energy distribution of the light passing through the aperture. In comparison to those recent developments, our work is based on the tracking of focal spots, offering high energy efficiency, high accuracy, calibration-free operation, noise tolerance (e.g., readout and shot noise), and fabrication-error tolerance. As it only requires a single intensity measurement without any preconditions for specimen or illumination, the meta SHWFS holds great potential in the development of portable and point-of-care devices for early-stage diagnosis, endoscopic live cell imaging, and on-site industrial inspection.

## Materials and methods

### Preparation of meta SHWFS

Meta SHWFS consisted of a silicon nitride rectangular cuboid arranged on a subwavelength square lattice with a periodicity of *U* = 350 nm. The width of each metaatom is precisely controlled within a range from 60 nm to 275 nm to achieve 2π phase coverage within a height of 630 nm at a wavelength of 532 nm. The phase delay imparted by the metaatoms were retrieved by using rigorous coupled-wave analysis (Supplementary Note 4 in the Supplementary Information). The meta SHWFS was fabricated on 630 nm thick silicon nitride on 500 μm thick fused silica. Silicon nitride film was deposited by plasma-enhanced chemical vapor deposition. The sample was spin-coated with a 300 nm thick positive electron beam resist (AR-P 6200) and the pattern was generated using electron beam lithography. After development, an electron-beam-evaporated aluminum oxide layer was used to reverse the generated pattern using a lift-off process and was used as a hard mask for dry etching of the underlying silicon nitride layer. The dry etching was performed using an inductively coupled plasma reactive ion etching process. The aluminum oxide layer was then dissolved in buffered oxide etchant. Figure S5 in the Supplementary Information shows the fabrication flow.

### Large angle calibration of meta SHWFS

Figure 2a shows schematics of the optical setups. A single-mode laser (λ = 532 nm) was used to characterize the meta SHWFS. Two 1-axis Galvano mirrors were used to control the angle of incidence. Each mirror is located, following a 4*f* relay system. Meta SHWFS is positioned in a conjugate plane of the second Galvano mirror. The focal spots of meta SHWFS were measured using an imaging system with an objective lens (Olympus; UPlanXAPO 4×, NA = 0.16). The effective pixel size of 0.4625 μm, which is comparable to the pixel size of recently developed CMOS sensors, was accomplished through a 4× imaging system with a physical pixel size of 1.85 μm (FLIR Blackfly S BFS-U3-120S4M-CS).

### Supplementary information

Supplementary information
