# Supplementary material for: Meta Shack–Hartmann wavefront sensor with large sampling density and large angular field of view: phase imaging of complex objects
Source: Light Sci Appl. 2024 Aug 12;13:187. doi: 10.1038/s41377-024-01528-9 (PMC11319597; doi:10.1038/s41377-024-01528-9)
Supplement: Supplementary file 1 — Supplementary information [file 41377_2024_1528_MOESM1_ESM.docx]

**Supplementary** **Information for**

**Meta Shack-Hartmann wavefront sensor**

**with large sampling density and large angular field of view:**

**Phase imaging of complex objects**

Gi-Hyun Go, Dong-gu Lee, Jaeyeon Oh, Gookho Song, Doeon Lee & Mooseok Jang^*^

Department of Bio and Brain Engineering, Korea Advanced Institute of Science and Technology (KAIST), Daejeon 34141, Republic of Korea

Corresponding authors: * mooseok@kaist.ac.kr

**The official email addresses of all authors:**

| Gi-Hyun Go: | gh_go@kaist.ac.kr |
| --- | --- |
| Dong-gu Lee: | dong-gu@kaist.ac.kr |
| Jaeyeon Oh: | jaeyeon7873@kaist.ac.kr |
| Gookho Song: | gookho.song@kaist.ac.kr |
| Doeon Lee: | leedoun@kaist.ac.kr |
| Mooseok Jang: | mooseok@kaist.ac.kr |

**The full contact details of the corresponding author:**

| Address | 1117, CMS Bldg. (E16), 291, Daehak-ro,  Yuseong-gu, Daejeon, 34141, Korea |
| --- | --- |
| Telephone | +82-42-350-4328 |

**Supplementary Text**

In the main text, we have reported the meta Shack-Hartmann wavefront sensor (SHWFS) with a large sampling density and large angular field of view. In this supplementary information, we present the details of the results of simulations and experiments.

**Supplementary Note 1. Principle of the meta SHWFS**

The phase of each metalens is described as the hyperbolic phase:

|  | $\psi_{\mathrm{lens}}\left( x,y \right)=-\frac{2\pi}{\lambda}\left( \sqrt{f^{2}+x^{2}+y^{2}}-f \right)$ | (1) |
| --- | --- | --- |

where *λ* is the wavelength of incident light, *x* and *y* are the coordinates of the meta-atoms, and *f* is the focal length of the metalens. When incident light strikes the metalens at an angle *θ*, the phase of light after the metalens becomes approximately as:

| $\psi=\psi_{\mathrm{lens}}+kx\sin\theta\approx-\frac{2\pi}{\lambda}\sqrt{f^{2}+\left( x-ftan\theta_{x} \right)^{2}+\left( y-ftan\theta_{y} \right)^{2}}+const$ | (2) |
| --- | --- |

where the subscripts *x* and *y* of *θ* refer to the components in the corresponding directions. As a result, the effect of oblique incidence is a translation of the focal spot as:

|  | $D_{i}=ftan\theta_{i} (i=x,y)$ | (3) |
| --- | --- | --- |

If the diameter of metalens is sufficiently smaller than the spatial frequency of the phase profile, the incoming light on each metalens can be approximated to the plane wave with local incident angle *θ*. In this case, the local phase gradient on the metalens is related to the local incident angle, following the generalized Snell’s law:

|  | $\partial_{i}\phi=ksin\theta_{i} (i=x,y)$ | (4) |
| --- | --- | --- |

From the equation (3) and (4), the phase gradient can be achieved from the displacement of focal spot as:

|  | $\partial_{i}\phi=ksin(\tan^{-1} \frac{D_{i}}{f}) (i=x,y)$ | (5) |
| --- | --- | --- |

Finally, the phase can be achieved by integrating the local phase gradient values measured at each lenslet.

**Supplementary Note 2. Parameter design to maximize the number of resolvable angles**

We determined the parameter of meta SHWFS to maximize the number of resolvable angles $N_{\theta}$. For this purpose, the root mean square of the localization error $\Delta_{\mathrm{res}}$ is calculated for simulated focal spots. The focal spots are generated at some positions convolved with a point spread function (PSF):

|  | $PSF=\left( \frac{2J_{1}(v)}{v} \right)^{2}$ | (6) |
| --- | --- | --- |

where J_1_ is the Bessel function of the first kind of order 1, v=(2πNA*r*)/*λ* and *r* is the radial coordinate. The position of the focal spot is determined with subpixel accuracy through the calculation of the radial symmetry center (details in Supplementary Note 3). Figure S1a shows the localization error $\Delta_{\mathrm{res}}$from the simulation for different signal-to-noise ratios (SNR). For all SNRs, $\Delta_{\mathrm{res}}$ decreases until the focal length increases up to 40 μm (left). For larger focal lengths than 40 μm, $\Delta_{\mathrm{res}}$ increases as the focal length increases (right). For the short focal length, the spot size is smaller than a single pixel. Therefore, it becomes impossible to accurately localize the spot with subpixel resolution. Conversely, for the long focal length, the spot size is significantly larger than the spot size. Many noisy pixels in the spot hinder the accurate tracking of the centroid position. We determined the focal length to be 30 μm to maximize $N_{\theta}={(2\Delta_{\max}/\Delta_{\mathrm{res}})}^{2}$ for all SNR range (Figure S1b). Specifically, we can obtain the localization error $\Delta_{\mathrm{res}}$ of $0.13 \mu m$ and the number of resolvable angles $N_{\theta}$ of 3600 for SNR=10 dB.


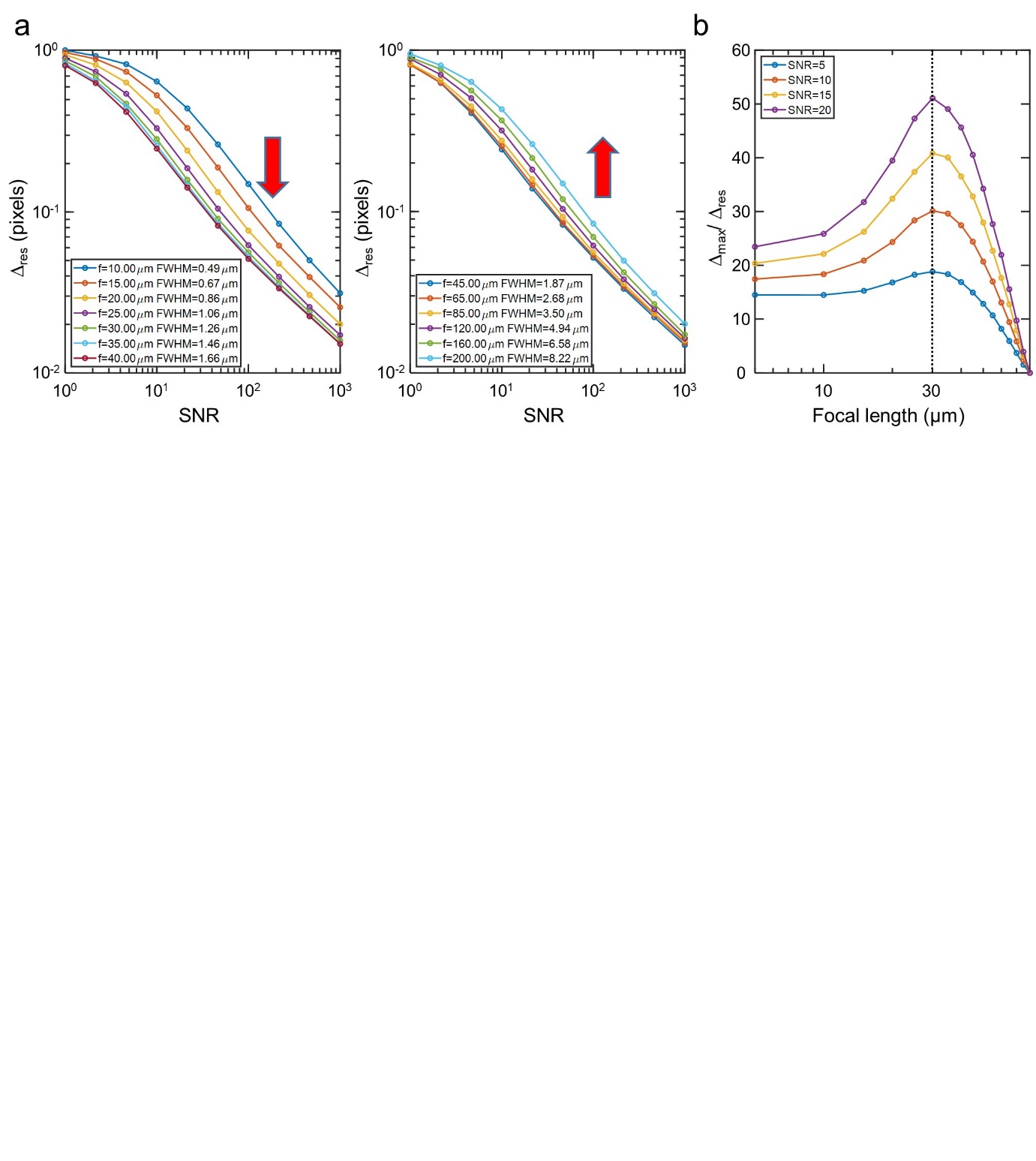


**Figure S1. a metalens SHWFS.** **a** Root mean square error $\Delta_{\mathrm{res}}$ of spot tracking versus the SNR for different focal lengths. **b** $\Delta_{\max}/\Delta_{\mathrm{res}}$ versus the focal length for different SNRs.

**Supplementary Note 3. Focal spot positioning using radial symmetry center**

In this study, the subpixel accuracy in positioning the focal spot is accomplished through the utilization of the radial symmetry center algorithm, specifically developed for particle tracking. This approach attains tracking accuracies close to theoretical limits and exhibits notably faster execution times compared to Gaussian fitting, facilitated by an analytic and non-iterative calculation. Figure S2 shows the key principle of the algorithm.

Let a discretely sampled intensity $I_{i,j}$, where subscripts label rows and columns pixels of the detector. At midpoint ($x_{k},y_{k}$) of four pixels denoted as $\{\left( i,j \right),\left( i+1,j \right),\left( i,j+1 \right),(i+1,j+1)\}$, intensity gradient can be obtained as:

|  | $\vec{\nabla}I=\left[ \begin{matrix} 1 & -1 \\ 1 & 1 \end{matrix} \right]\left[ \begin{matrix} I_{i+1,j+1}-I_{i,j} \\ I_{i,j+1}-I_{i+1,j} \end{matrix} \right]=\left[ \begin{matrix} \left( I_{i+1,j+1}-I_{i,j} \right)-\left( I_{i,j+1}-I_{i+1,j} \right) \\ \left( I_{i+1,j+1}-I_{i,j} \right)+\left( I_{i,j+1}-I_{i+1,j} \right) \end{matrix} \right]$ | (7) |
| --- | --- | --- |

Thus, the slope of the gradient at the midpoint can be obtained as:

|  | $m_{k}=\frac{\left( I_{i+1,j+1}-I_{i,j} \right)+\left( I_{i,j+1}-I_{i+1,j} \right)}{\left( I_{i+1,j+1}-I_{i,j} \right)-\left( I_{i,j+1}-I_{i+1,j} \right)}$ | (8) |
| --- | --- | --- |

The center of the focal spot ($x_{c},y_{c}$) is determined by identifying the point where the lines of gradient slope at all midpoints intersect, owing to the radial symmetry. Therefore, the algorithm determines the center position that minimizes $\sum_{k} d_{k}^{2}w_{k}$, where $d_{k}$ is the distance between the focal spot center and the line of gradient slope, and $w_{k}$ is some weighting of point ($x_{k},y_{k}$).

|  | $d_{k}^{2}=\frac{\left[ \left( y_{k}-y_{c} \right)-m_{k}\left( x_{k}-x_{c} \right) \right]^{2}}{m_{k}^{2}+1}$ | (9) |
| --- | --- | --- |
|  | $w_{k}=\frac{\left\vert\vec{\nabla}I_{k} \right\vert^{2}}{d_{c}}$ | (10) |

where $d_{c}$ is the distance between ($x_{c},y_{c}$) and the centroid of $\left| \vec{\nabla}I_{k} \right|$.

After some algebra, the center position ($x_{c},y_{c}$) can be derived as:

|  | $x_{c}=\xi^{-1}\left[ \left( \sum_{k} \frac{m_{k}w_{k}\left( y_{k}-m_{k}x_{k} \right)}{m_{k}^{2}+1} \right) \left( \sum_{k} \frac{w_{k}}{m_{k}^{2}+1} \right)-\left( \sum_{k} \frac{m_{k}w_{k}}{m_{k}^{2}+1} \right) \left( \sum_{k} \frac{w_{k}\left( y_{k}-m_{k}x_{k} \right)}{m_{k}^{2}+1} \right) \right]$ | (11) |
| --- | --- | --- |
|  | $y_{c}=\xi^{-1}\left[ \left( \sum_{k} \frac{m_{k}w_{k}\left( y_{k}-m_{k}x_{k} \right)}{m_{k}^{2}+1} \right) \left( \sum_{k} \frac{m_{k}w_{k}}{m_{k}^{2}+1} \right)-\left( \sum_{k} \frac{m_{k}^{2}w_{k}}{m_{k}^{2}+1} \right) \left( \sum_{k} \frac{w_{k}\left( y_{k}-m_{k}x_{k} \right)}{m_{k}^{2}+1} \right) \right]$ | (12) |
|  | $\xi=\left( \sum_{k} \frac{m_{k}w_{k}}{m_{k}^{2}+1} \right)^{2}-\left( \sum_{k} \frac{m_{k}^{2}w_{k}}{m_{k}^{2}+1} \right)\left( \sum_{k} \frac{w_{k}}{m_{k}^{2}+1} \right)$ | (13) |


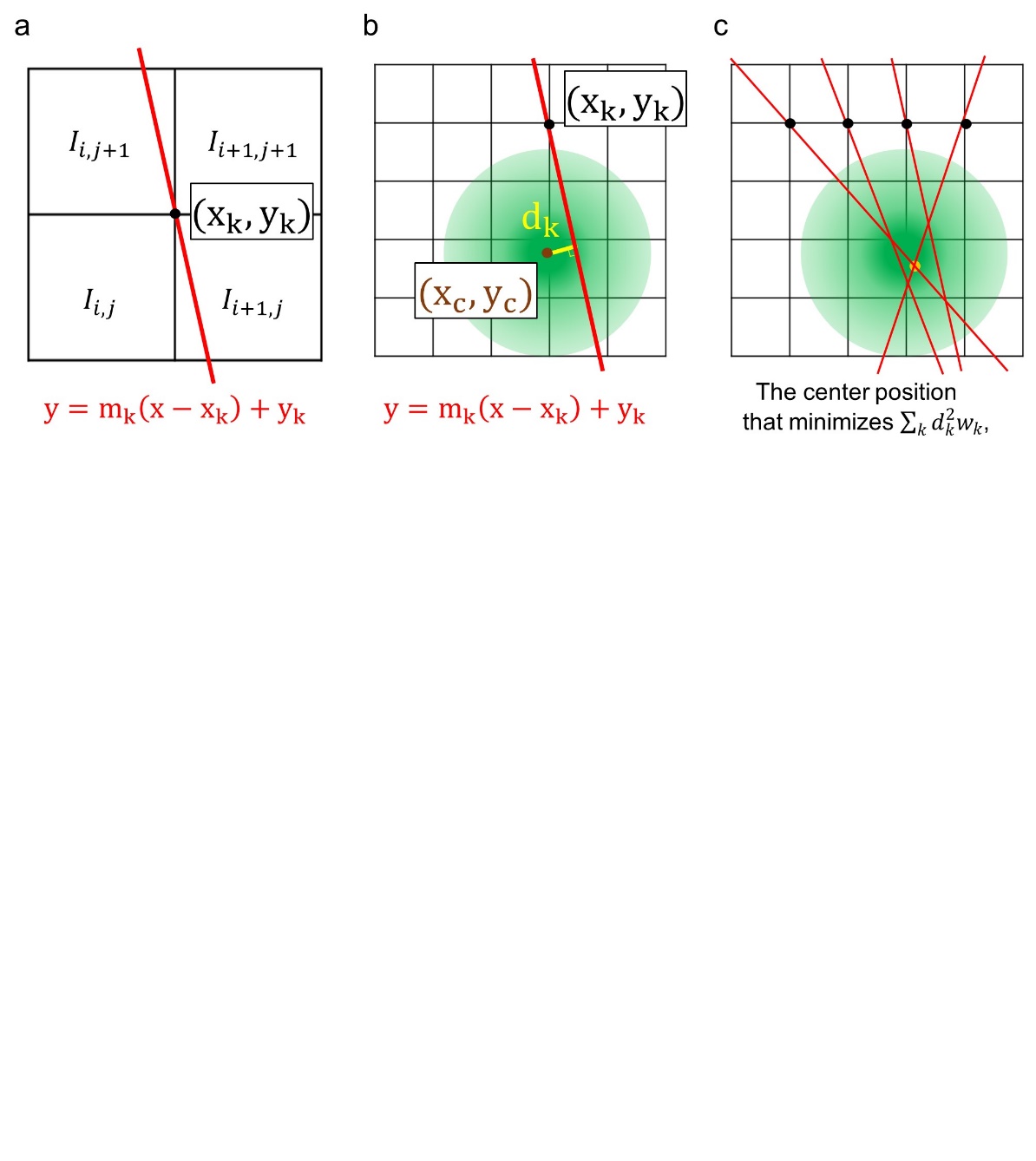


**Figure S2. Focal spot positioning using radial symmetry center. a** Intensity gradient at midpoint ($x_{k},y_{k}$) of four pixels denoted as $\{\left( i,j \right),\left( i+1,j \right),\left( i,j+1 \right),(i+1,j+1)\}$. **b** Calculation of the distance between the focal spot center and the line of gradient slope. **c** Center position that minimizes $\sum_{k} d_{k}^{2}w_{k}$, where $d_{k}$ is the distance between the focal spot center and the line of gradient slope, and $w_{k}$ is some weighting of point ($x_{k},y_{k}$).

**Supplementary Note 4. Phase and transmission of the SiN_x_ nanopost**

The meta SHWFS consists of silicon nitride (SiN_x_) nanoposts sitting on a fused silica substrate as shown in Figure S3a. Figure S3b shows the phase and transmission of the nanoposts calculated using a rigorous coupled wave analysis at a wavelength of 532 nm. In this study, we selected the width range from 60 nm to 275 nm to cover 2π phase modulation with high transmission. The widths of the nanoposts corresponding to the greyed regions in Fig. 2b correspond to high-quality-factor resonances and are excluded in the design of the metasurface.


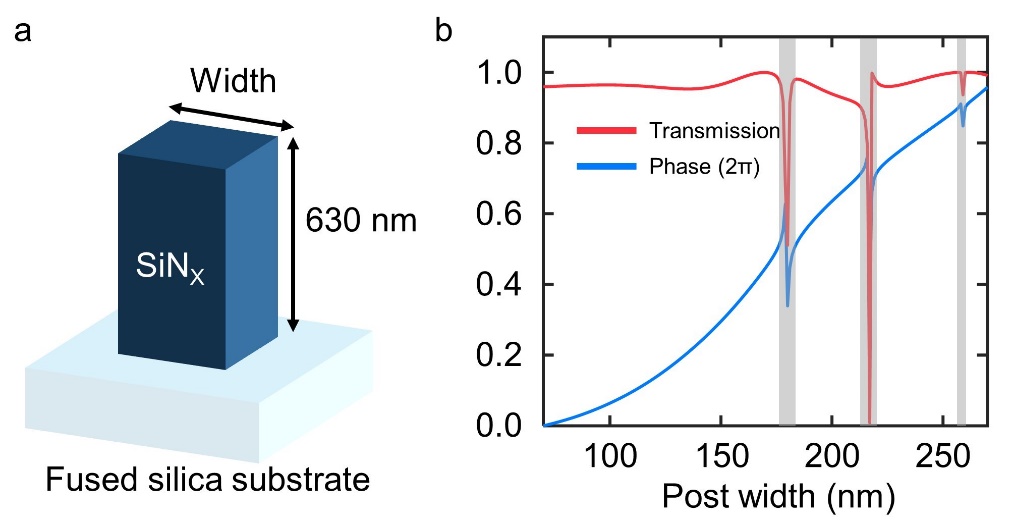


**Figure S3. Result of rigorous coupled-wave analysis. a** Silicon nitride (SiNx) nanoposts sitting on a fused silica substrate. **b** Transmission and phase imparted by a SiN_x_ nanopost for different widths.

**Supplementary Note 5. Fabrication process**

The meta SHWFS was fabricated on 630 nm thick silicon nitride on 500 μm thick fused silica. Silicon nitride film was deposited by plasma-enhanced chemical vapor deposition. The sample was spin-coated with a 300 nm thick positive electron beam resist (AR-P 6200) and the pattern was generated using electron beam lithography. After development, an electron-beam-evaporated aluminium oxide layer was used to reverse the generated pattern using a lift-off process and was used as a hard mask for dry etching of the underlying silicon nitride layer. The dry etching was performed using an inductively coupled plasma reactive ion etching process. The aluminium oxide layer was then dissolved in buffered oxide etchant. Figure S4 shows the fabrication flow.


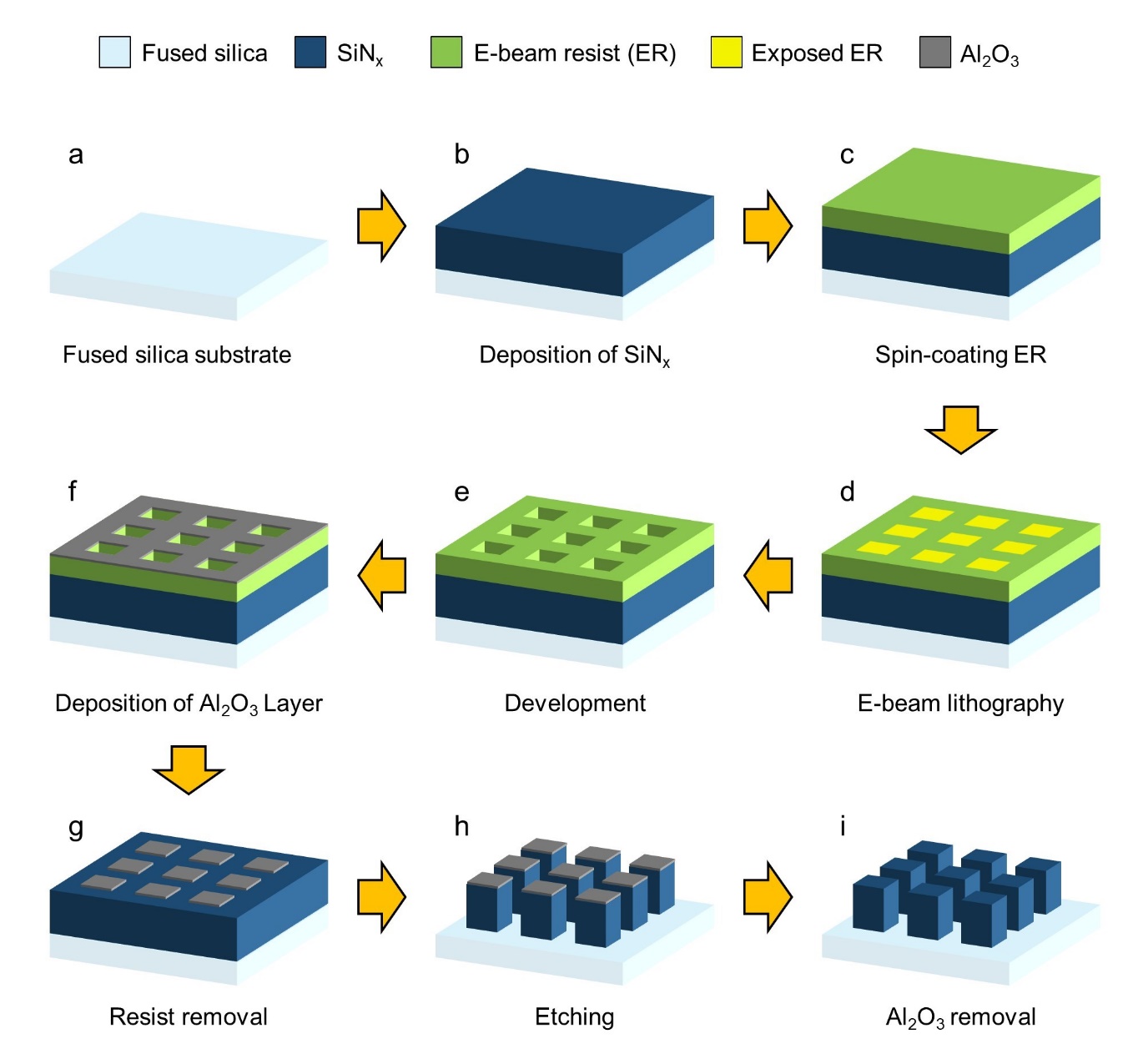


**Figure S4. Flow for fabrication of a metalens SHWFS.** **a-b** Plasma-enhanced chemical vapor deposition of silicon nitride (SiN_x_) on a fused silica substrate. **c** Spin coating of positive E-beam resist (AR-P6200) with a thickness of 300 nm. **d** Pattern generation through electron beam lithography. **e-g** Electron beam evaporation of aluminium oxide (Al_2_O_3_) layer and a lift-off process to form the hard mask. **h** Dry etching through an inductively coupled plasma reactive ion etching process. **i** Removal of the aluminium oxide layer.

**Supplementary Note 6. Alignment protocol of meta SHWFS**

We carefully aligned our meta SHWFS. As our meta SHWFS was created using nano fabrication techniques, the lens pitch was precisely set to 12.95 μm. Thanks to this precision, near optimal performance predicted from theory can be guaranteed only based on careful alignment between metalens array and sensor, whose procedure is detailed below. Figure S5a depicts the experimental setup for the alignments. All alignments are based on the focal spots of the metalenses at four corners (Figure S5b). The detailed process is outlined as follows:

1. **Tilt alignment and axial position:** We observed the focal spots of the four metalenses and record the peak intensities while moving the metalens array along the vertical direction. Due to the extremely short focal length of the metalens, the relative axial positions at the four corners are achieved with micrometer-scale accuracy. Therefore, the tilt angle of the metasurface can be adjusted with an accuracy of approximately $\tan^{-1} [1 \mu m/1.295 mm]\sim0.04^{\circ}$.
2. **Effective sensor pixel size and lens pitch:** Because the lens pitch size can be assumed to be precisely 12.95 μm, the spacing between the four spots is 99 × 12.95 μm. Therefore, the effective pixel size is obtained by dividing 99 × 12.95 μm by the number of sensor pixels between the spots. To achieve $P_{\mathrm{eff}}$ = 0.4625 μm, the size of metalens is equal to 28 × 28 pixels. Therefore, we carefully adjusted the imaging system so that the distance between two spots is 99 × 28 pixels. This method accurately controls the effective pixel size, because even a mere 1% deviation can result in the 99th lens or empty space being observed at the opposite corner. As the focal spot positions can be determined with sub-pixel resolution, the error in effective pixel size achievable is (28 × 99)^-1^=0.04%.


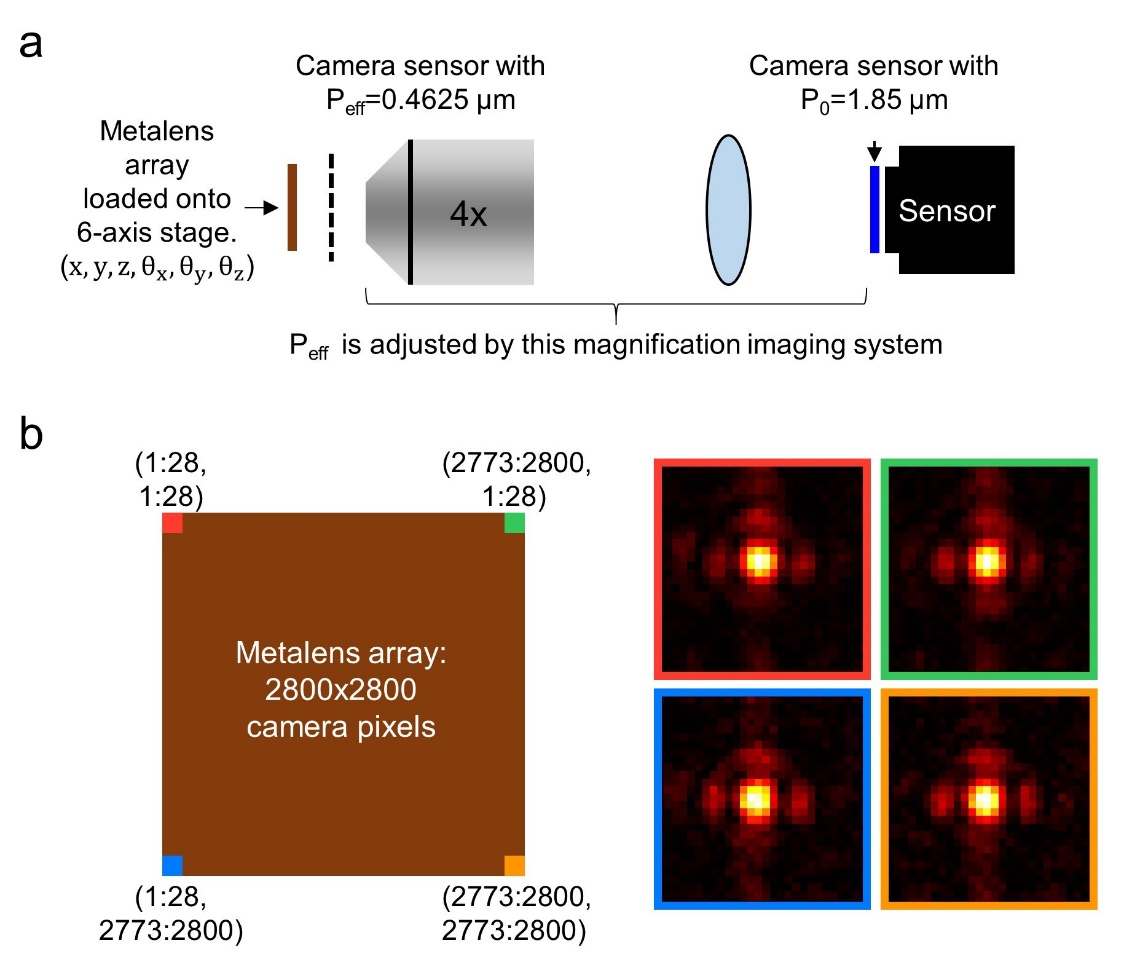


**Figure S5. Alignment and calibration of the meta SHWFS. a** Experimental setup for the calibration of the meta SHWFS. **b** Four focal spots of the four metalenses at four corners. The numbers outside the left image denote the camera pixels of each area.

**Supplementary Note 7. FDTD analysis on chromatic aberration & its impact on centroid tracking**

Since our metalens is designed for 532 nm, chromatic aberration can occur. This aberration arises from dispersion within the periodic lattice, akin to Fresnel lenses, resulting in different focal lengths as: $f=f_{c}\lambda_{c}/ \lambda$, where the center wavelength and focal length are $\lambda_{c}=532 nm$ and $f_{c}=30 \mu m$, respectively. Due to the extremely short focal length of our metalens, the shift in focal length is limited to a few micrometers, even across the wide wavelength range from 432 nm to 632 nm. Because this change in focal distance is smaller than the depth of field, $DOF=\lambda/\left[ 2\left( 1-\cos\theta\right) \right]=11.8 \mu m$, centroid tracking is feasible (Figure S6a).


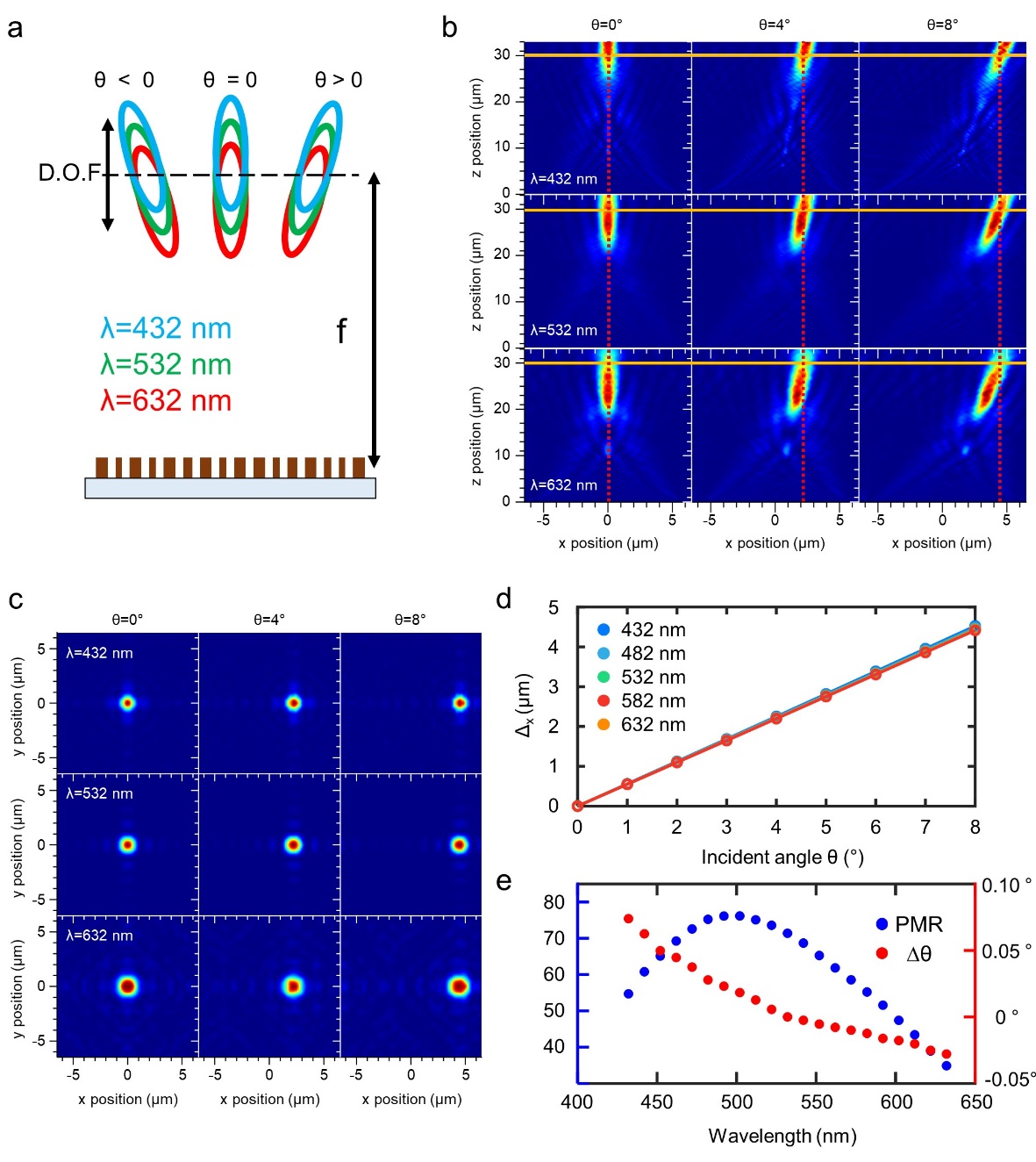


**Figure S6.** FDTD simulation results of metalens for different wavelengths. **a** Schematic image for chromatic aberration with small focal length. **b** Intensity images in the xz-plane for different wavelengths. The orange line indicates the sensor plane (z=30 μm). The red dotted line represents the central position for λ_c_=532 nm. **c** Intensity images at the focal plane for different wavelengths. **d** Results of the focal spot racking. **e** Peak-to-mean ratio (PMR) and angle error for different wavelengths.

We have performed numerical analysis on chromatic aberration with finite-difference time-domain (FDTD) simulation over the wavelength range of 432 nm - 632 nm. Through the numerical analysis, we confirmed that the focal spots for different wavelengths are virtually undistorted and move the same distance. Figure S6b depicts the focal spots for different wavelengths – 432 nm, 532 nm, and 632 nm, in the xz-plane. The results are similar to the schematic image depicted in Figure S6a where focal spot displacement along optical axis is significantly smaller than axial spot size. Figure S6c shows the intensity images at the focal plane for the different wavelengths. Figure S6d illustrates the results of the centroid tracking for the focal spots, presenting nearly identical tracking results for the wavelength range of 432 nm - 632 nm. For further analysis, Figure S6e presents the peak-to-mean ratio (PMR) values and the angle error $\Delta\theta$ (i.e. the deviation of the measured angle from that of the central wavelength) at varying wavelengths. As expected, the differences of $\Delta\theta$ for different wavelengths are confined within ± 0.1°, indicating that the proposed meta SHWFS is directly usable in the broad range of source wavelength. The PMR values decrease from around 80 to 40 as the wavelength becomes more deviated from the central wavelength of 532 nm. The reduced value of PMR would make the centroid tracking more vulnerable to sensor and shot noises. However, as presented in Figure S1, the centroid tracking is robustly working for the SNR of 10. In summary, due to its short focal length, the impact of achromatic aberration remains marginal, enabling the use of the proposed meta SHWFS in the entire visible spectrum of 432 nm - 632 nm.

**Supplementary Note 8. Phase imaging with different** $\boldsymbol{N}_{\boldsymbol{\theta}}$ **values**

To examine the effect of the number of resolvable angles $N_{\theta}$, we examined the quality of reconstructed the phase with different $N_{\theta}$ values. For this purpose, we prepared the ground truth image of the colon tissue obtained by off-axis holography reconstruction algorithm. We calculated the wavefront slope calculated of the colon phase, and mapping the wavefront slope to the basis set [$\theta_{x}$, $\theta_{y}$] with different $N_{\theta}$ values (Figure S7a). Figure S7b shows the reconstructed phase image from the wavefront slopes using different basis sets with $N_{\theta}$=3600 and 400 for maximum acceptance angle $\theta_{\max}$=8°. We confirmed that the reconstructed phase image with $N_{\theta}$=400 is distorted, making it impossible to discern details in the colon tissue, while phase image with $N_{\theta}$=3600 exhibits sufficient quality to examine all detailed features. These results show that the degree of freedom in measuring wavefront angle, $N_{\theta}=3600$ with the angular range of ± 8° is sufficient for the phase imaging of complex phase object.


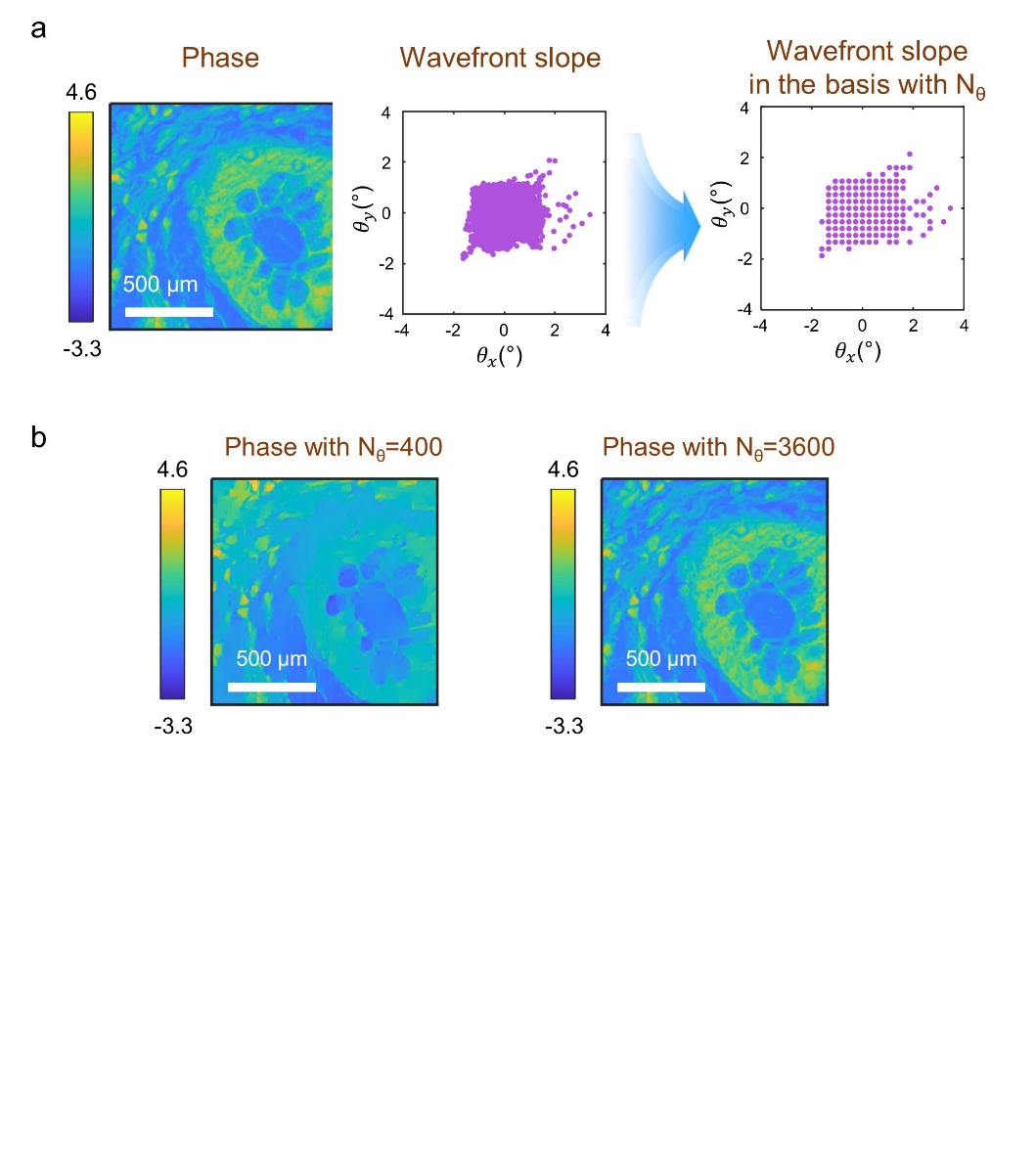


**Figure S7. The effect of the number of resolvable spot positions. a** Mapping process of the wavefront slope to the basis with different $N_{\theta}$ values. **b** Reconstructed phase image with different $N_{\theta}$ values.

**Supplementary Note 9. Chromatic aberrations of meta SHWFS**

The focusing efficiency of our metalens was experimentally measured to be 47.7%. Here, the focusing efficiency is defined as the fraction of the light in the focal plane with a radius equal to three times the full width at half maximum (FWHM) of the focal spot. To investigate the effect of lens size, finite-difference time-domain (FDTD) simulations were conducted on metalens arrays with varying lens sizes while maintaining a constant numerical aperture (NA) of 0.21. Figure S8a and b illustrate the focal spots and their line profiles generated by the metalenses. As the size of the metalens decreases, the ratio of focal spot size to lens size increases, resulting in a smaller peak-to-mean ratio (PMR) and the severe cross-talk from adjacent metalenses, as shown in Figure S8c. Consequently, accurate centroid tracking becomes challenging. Figure S8d represents the results of the centroid tracking for the focal spot at different incident angles. Due to the low PMR and large cross-talk effects, tracking accuracy significantly decreases for metalenses smaller than 6 μm.


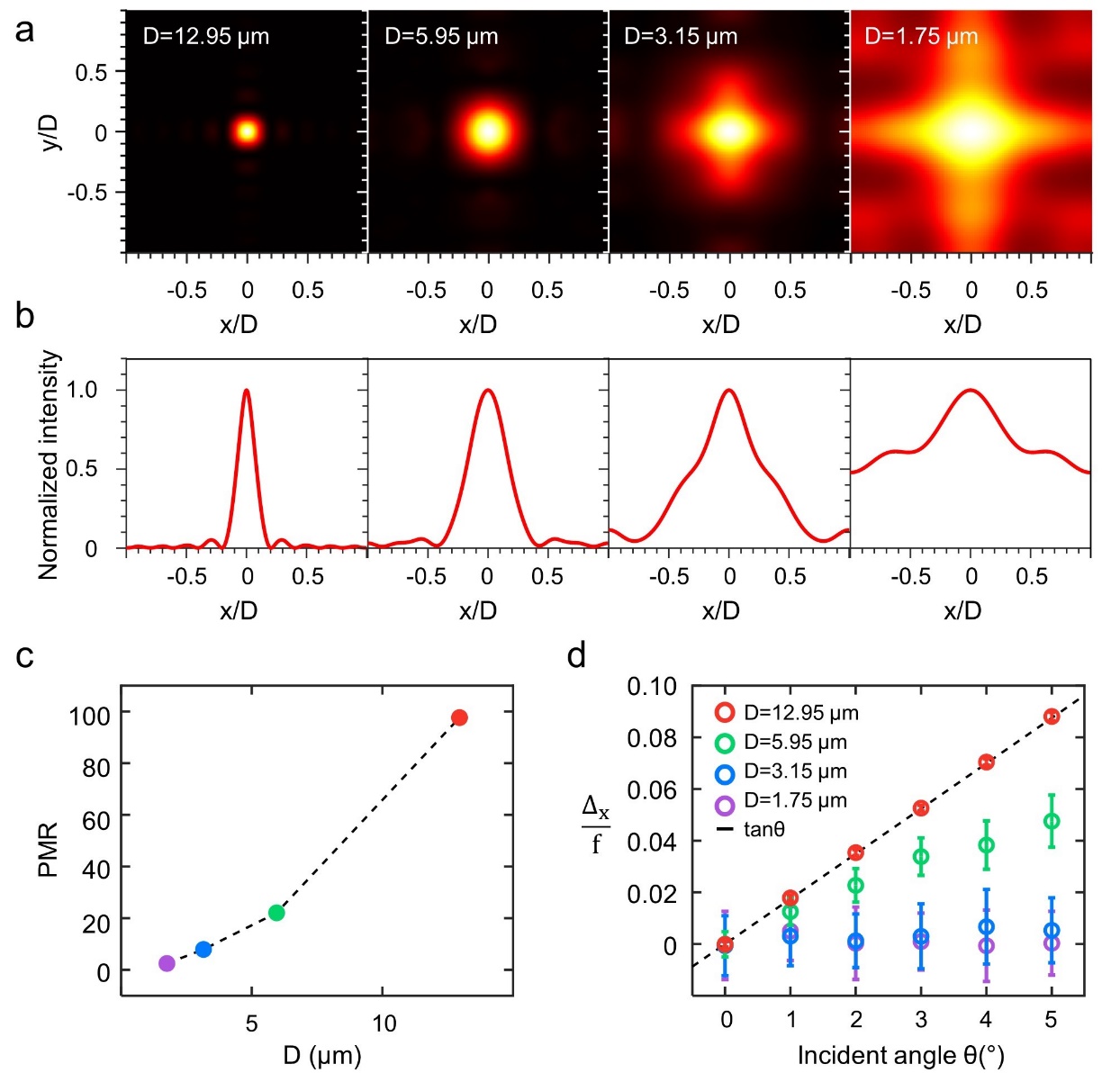


**Figure S8.** The effect of lens size. **a** Focal spots calculated through finite-difference time-domain (FDTD) simulations for different lens sizes and **b** their line profiles. **c** Peak-to-mean ratio (PMR) of the focal spots. **d** Results of the centroid tracking.

**Supplementary Note 10. Potential strategies for further improvement**

In general, the dynamic range decreases as the lens size decreases. The dynamic range is qualitatively proportional to the ratio of lens size to focal spot size. We examined the potential strategies to mitigate the trade-offs between the lens size and the dynamic range. The first solution is the use of a meta SHWFS with subgroups composed of four different multifocal metalens (depicted as 1, 2, 3, 4), as shown in Figure S9a. In conventional SHWFS configurations, a focal spot produced by a certain lens (orange area) should be confined within the lens size because the focal spot is indistinguishable from the focal spots of adjacent metalenses. However, if the focal spots of each lens are distinguishable, the focal spot can cover the red square region, capable of approximately doubling the dynamic range. We designed a lens capable of distinguishing focal spots based solely on their spatial deployment, without relying on relative intensity information. This design allows for application beyond phase targets, extending to general samples where intensity variations may occur. A metalens generating three or fewer spots is insufficient to make the focal spots indistinguishable. For example, the three focal spots created by a lens ‘A’ and the one of the focal spots from the other three lenses can overlap one by one at the same location. In this case, it is difficult to know the presence of the focal spots from lens ‘A’. Relative intensity is only useful for the phase targets. Therefore, we designed the metalenses generating four focal spots where the spots created by two different lenses can intersect at only one point. Let’s denote the spots from each lens as S_1_, S_2_, S_3_, and S_4_, respectively. Figure S9b shows the simulation results of focal spots of multifocal metalenses. The lower row depicts the phase profiles generated by Gerchberg-Saxton algorithm. Figure S9c shows the examples of the overlap of the focal spots. We can always distinguish each other regardless of how they overlap.


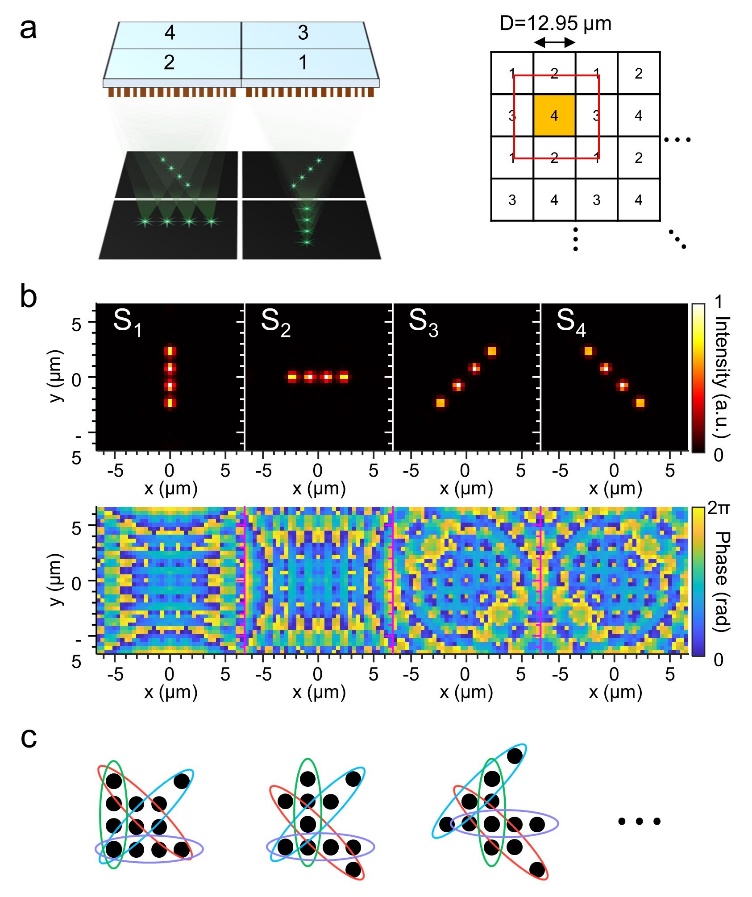


**Figure S9. a** Concept of meta SHWFS with subgroups composed of four different multifocal metalenses. **b** Four different patterns generated by multifocal metalenses. **c** Examples of the overlap of focal spots.

Alternatively, in case that one may want a single focal spot, particularly for applications involving weak signal beams or low sensor sensitivity, meta SHWFS added a local constant phase to each lens in the form of a checkerboard pattern is available to increase the dynamic range. In the SHWFS applications, the cross-talk is severe when the two focal spots close to each other (Figure S10a). In the case of a partially incoherent light with $\Delta\lambda$ < 22 nm (such as a typical monochromatic LED), the two focal spots cause interference because the coherence length exceeds D. Therefore, meta SHWFS added constant phase π with a checker board pattern (Figure S10b) can induce destructive interference between two focal spots. Consequently, the two spots can be separated for a large incident angle $\theta$. Figure S10c and d respectively show the focal spot behaviors simulated for both cases, with and without local phase. With the local phase, the sub-peaks between the focal spots are reduced at the incident angles of 5° and 7°. The difference is even more pronounced at $\theta$=11°. Without a local phase, the two spots merge and become completely indistinguishable, whereas with a local phase constant, the two spots can still be distinguished. Figure S10e illustrates the results of central tracking. In case without a local phase constant, accurate tracking is not feasible due to cross-talk, and tracking fails for large angles. Conversely, employing a local constant phase enabled precise tracking up to 11° as cross-talk reduced due to destructive interference.


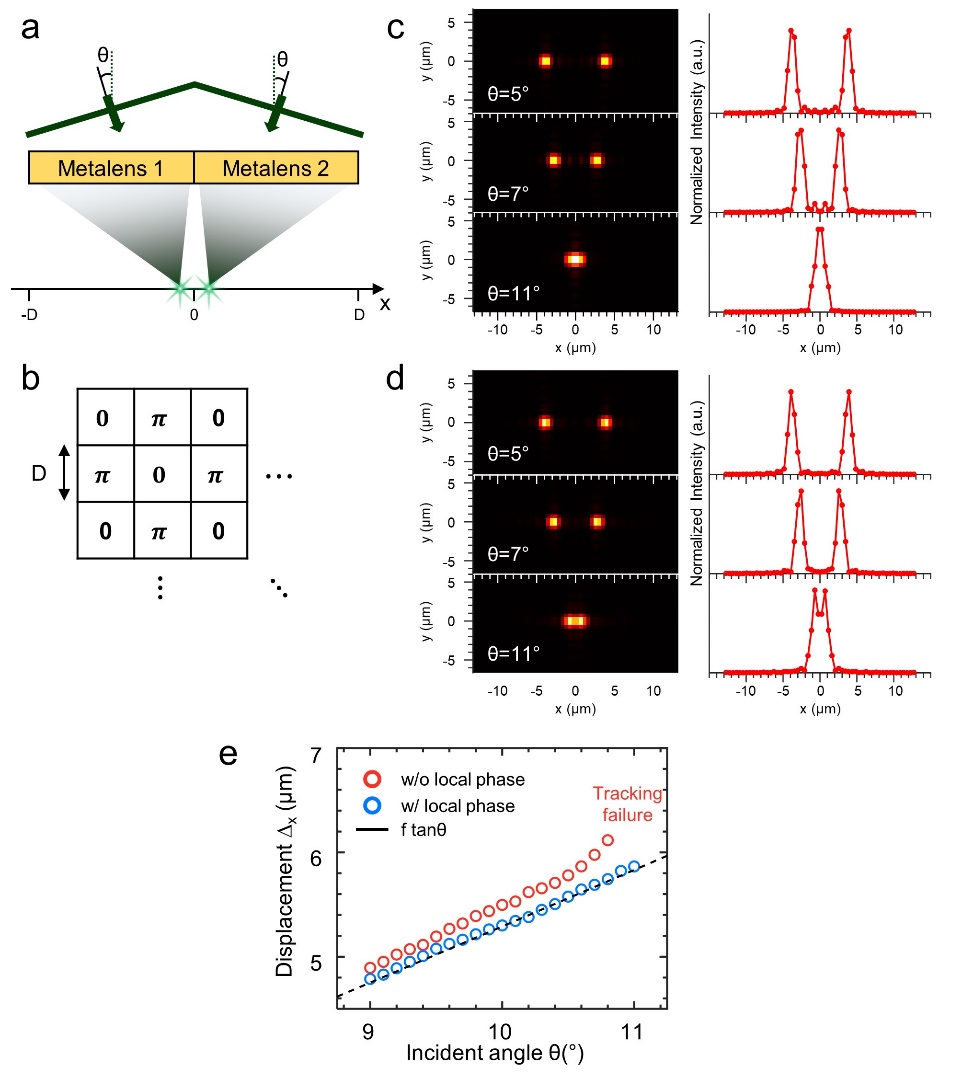


**Figure S10.** **Meta SHWFS added a local constant phase**. **a** Cross-talk caused by two focal spots. **b** Constant phase π added in the form of a checker board pattern. **c-d** Focal spot behaviours simulated for both cases, with and without local phase. e Results of the focal spot tracking.
